# The Multiplication of Dental Associations

**Published:** 1902-11

**Authors:** 


### THE MULTIPLICATION OF DENTAL ASSOCIATIONS.

In the October number the subject of "Organizations, Present and Future" was considered in relation principally to national organizations, but that editorial failed to exhaust the subject, and possibly even a series of papers might equally fail to meet all that would be required to satisfy the most exacting. Indeed, it may be assumed that individualization will, in all future time, bar the way to an agreement as to the best course to pursue in national, State, or local organizations. One set of persons view the social side as of great importance as a method of drawing individuals together and insuring a large attendance, hence every meeting begins or ends with a menu of supposed good things. There can be no valid objection to this method if the tickling of the gustatory faculty is not carried to the extent of making it primary and the mental feast a secondary consideration. To the man who attends meetings not for that which he may procure in the way of eatables, but for that mental exhilaration that comes to him through attrition with other minds, this method will not appeal; indeed, he may regard it as an evidence of degeneration in society work. To the writer it appears as a marked indication of a loss of scientific interest and as a tacit acknowledgment that dentists have reached a period when papers and discussions upon important topics have ceased to draw. It is feared this conception of the dental condition is unfortunately based on fact. The thought has unwillingly been forced upon the writer's mind that the organization briefly alluded to in the last number is bound to come before this century closes, an organization in which all the members will be influenced by one purpose,—that of advancement of the scientific side of the profession,—and this must necessarily include all the branches known under the name of dentistry. Such an organization can have no use for, and will not admit a place for, any menu, however enticing, nor will it be drawn away from the object of the gathering by outside inducements.

The tendency to separation is becoming more pronounced yearly, and is being fostered, largely aside from the banqueting method, by the fact that persons desirous of improving in special work, or in dentistry as a whole, have found it necessary to organize separate societies and confine their work to a limited area of thought and practice. Hence, while national, State, and local societies were considered amply sufficient a few years ago, and certainly did suffice for the needs of dentistry at that period, there came a natural reaction, an opposition to this generalization and commingling of subjects related, and yet in a measure antagonistic.

The first departure came when the dental colleges found it was necessary to organize independently of all other dental associations and to arrange for their own well-being, which could not be accomplished in a national body as then organized. Then followed the formation of the Section on Stomatology of the American Medical Association, the members of which devote their time to the discussion of scientific topics, so called, and have nothing in common with the man interested in the practical details of his profession.

The formation of the association known as the "Institute of Dental Pedagogics" is another evidence of this segregating influence. It would seem that the National Association of Dental Faculties might very readily have covered all the subjects that this body meets annually to consider, but those interested in teaching problems thought otherwise, and a quite important body meets yearly with an enthusiasm that speaks well for the self-sacrificing spirit of the members and promises to be a source of great benefit to dental teaching in the future, as it has in the past.

The division of dentistry into specialties has resulted already in an organization of the "American Society of Orthodontists." This has had its second annual meeting this October in Philadelphia. It is not many years since the regulating of teeth was regarded as one of the least important of the operations the dentist was called upon to perform. Indeed, the dentist of the olden period held this work to be the most distasteful of all his proceedings. This now is all changed, thanks to the persevering efforts of Kingsley, Farrar, Angle, Jackson, and other advanced minds, and it has become the most important of all operative procedures, necessitating, in the opinion of some, a combination of all those interested in this work in a special organization. If this body will avoid the danger of regarding special methods as most important, to the neglect of principles underlying all operations in orthodontia, it must become a power for good in moulding practice. So far as the observations of the writer justifies an opinion, this seems to be the governing motive, and if this be continued one more organization has been founded to the advantage of professional work.

The question may be asked, however, Where will this end? Are we to have, in the future, the crown- and bridge-worker meeting, as a selected body to consider ways and means for the improvement of that method of practice? Will the prosthetic devotee eventually regard the Institute of Dental Pedagogics as too slow for the progress of his specialty, with the result of an association of mechanical dentists being formed? These questions will, in all probability, find an answer in the near future.

The entire subject is one of interest to the active professional man, and not without some cause for anxiety. The effect of this multiplication of societies has yet to be experienced. It seems as though the result should be to the advantage of dentistry as a whole, yet when we come to examine into the effect produced through the division of medicine into specialties, the result is not encouraging to the average layman, and very possibly not to the broad-minded professional worker. Instead of the patient securing, as formerly, a more or less intelligent opinion from his family doctor upon a given ailment, he is now sent from "pillar to post," each one giving, it may be, the opinion that the case belongs to some one else, until, finally, the patient may consider himself or herself fortunate if, between the divided responsibility, he or she is not landed in the last resting-place for humanity. We are face to face with a similar but more limited condition of affairs. The shifting of responsibility has begun in earnest, and we have our special prosthetic workers; our crown- and bridge-workers; our nitrous gas specialists; our oral surgeons; our ceramic workers in inlays and "continuous gum;" and last, but not least, our orthodontists, and some of the leaders of this, the new-born of the specialties, insist on its practitioners giving up all other forms of practice and confining their labor exclusively to this work.

The effect which this multiplication of specialties, and consequently the organization of new and independent associations, will have upon general organizations remains to be seen, but in the opinion of the writer it will eventually be disastrous. The time and expense involved in attending meetings will be a serious matter for consideration to the average practitioner. He will soon begin to find it is not to his interest to attend the sessions of the general meeting. He drops out and ceases to come in touch with his fellows in other branches. As the years roll on he has practically become unfamiliar with the dental profession, and has ceased to be a Doctor of Dental Surgery, with all that this degree implies. This is already manifest in those who practise specialties in medicine. This is the serious side of this entire business. The increase of these organizations devoted to specialties in dentistry has already begun to produce this effect, and as time advances this must become more marked. The future alone can determine whether this will be for the good or ill of dentistry. Let us hope it may be for good, but in any event it is bound to divide dentistry into many parts, rendering the work of the colleges more important than at any previous period, for there alone can the man or woman be trained into a composite in which all the parts of the profession will be moulded into one, and through which a graduate is made to acquire sufficient of all the specialties to enable him to give an intelligent opinion and to direct to practical good results.

---

